# Publication in the Australian medical student journal is associated with future academic success: a matched-cohort study

**DOI:** 10.1186/s12909-022-03607-0

**Published:** 2022-07-30

**Authors:** Alexander Wilton, Hasitha Pananwala

**Affiliations:** Ryde Hospital, Sydney, Australia

**Keywords:** Medical student, Medical student journal, Academic research, Undergraduate research

## Abstract

**Background:**

Medical student journals (MSJs) help to introduce the fundamentals of academic research and publication to future doctors. It has recently been shown that MSJs can influence doctors’ future academic and professional success, however these findings have not been replicated in an Australian cohort. The aim of this study was to examine the association between publication in the Australian Medical Student Journal (AMSJ) and markers of a student’s future academic trajectory, including future publication, attainment of higher academic degree or entry into specialist training.

**Methods:**

Articles authored by medical students in the AMSJ from 2010 to 2015 were retrospectively identified. A list of these student authors was made, with university- and year- matched control students randomly selected from university graduation databases. For all students, data related to academic success were obtained from multiple sources including PubMed®, Google, university databases and author affiliation information from publications. A multivariable conditional logistic regression model was used to assess correlation between variables. The primary outcome measure was the number of postgraduate PubMed®-indexed publications. Secondary outcome measures included attainment of higher degree or faculty position. Clinical speciality was also recorded.

**Results:**

Fifty-five AMSJ authors (14 case reports, 17 original research, 21 review articles) from 14 Australian universities were included. Publication in the AMSJ was associated with future PubMed® indexed publications (OR 3.43, 95% CI 1.74–6.77, *P* < 0.001) and higher degree attainment (OR 4.05, 95% CI 1.99–8.22, *P* = 0.0001). AMSJ authors were also significantly more likely to enter into surgical training (OR 2.53, 95% CI 1.10–5.84, *P* = 0.029). A multivariable conditional logistic regression model demonstrated that publication in the AMSJ was predictive of future PubMed indexed publication, independent of higher degree or faculty position attainment (OR 2.56, CI 1.22–5.39, *P* = 0.01).

**Conclusion:**

We have shown that publication in a MSJ is associated with markers of academic success in an Australian cohort. PubMed®-indexed publications, attainment of a Masters degree, and entry in to surgical training were all significantly correlated to AMSJ publication. A conditional logistic regression model demonstrated that medical student publication in the AMSJ influences the number of future PubMed®-indexed publications, independent of major academic confounding variables.

**Supplementary Information:**

The online version contains supplementary material available at 10.1186/s12909-022-03607-0.

## Background

Throughout history, medical students have made major contributions to medical research [1]. Students often face significant difficulty when publishing in established medical journals, which can discourage them from pursuing their academic goals [2, 3]. To better engage students in clinical research and promote scientific curiosity amongst our future clinicians, medical student journals (MSJs) have been developed to bridge the gap between undergraduate and postgraduate research. To ensure the validity and quality of the published research, MSJs operate under similar peer-review processes as their mainstream counterparts. To account for the relative inexperience of the authors, the peer-review process remains ‘student-friendly’ and offers encouragement and guidance to contributors.

Recently, the overall quality of MSJs has been called into question [4]. As a result, an effort has been made to examine the utility of publication in a MSJ, with a recent study in New Zealand demonstrating a positive association between student publication in the New Zealand Medical Student Journal (NZMSJ) and future academic success [5].

As global and Australian numbers of physician-scientists have declined in recent years, various strategies have been employed to engage students and younger physicians in research [6–8]. This includes a concerted shift in Australia from traditional Bachelor of Medicine, Bachelor of Surgery (MBBS) to the more research focused Doctor of Medicine (MD) program [9]. Medical student participation in research has been shown to be associated with improved short- and long-term scientific productivity [5, 10, 11]. It remains unclear however whether MSJs present any benefit to students over publishing their work in traditional medical journals [3].

The Australian Medical Student Journal (AMSJ) is a student led publication launched in 2010 to provide a medium for Australian medical students to publish their work and foster the next generation of Australian medical researchers [12]. The journal publishes 2 to 3 times a year with each issue containing a mix of case reports, opinion pieces, literature reviews and original research. The aim of this study was to examine the association between publication in the AMSJ and markers of a student’s future academic trajectory, including future publication, attainment of higher academic degree or entry into specialist training.

## Methods

### Study design

A matched-cohort study design was used. The population was Australian medical students. The sample was all first-authors of AMSJ articles in a 5-year period. Authors were paired with university- and year-matched control students from Australian medical schools.

### Data collection

AMSJ authors were identified from the AMSJ’s public online database. Controls were randomly identified from public medical school databases using a random number generator (Microsoft Excel for Windows, Microsoft Corp). Two control students were identified per AMSJ author. Control students were checked against the AMSJ database to ensure these students had not themselves published in the AMSJ. Outcome measure data was obtained using Google searches, university websites and author affiliation information from published articles. All data was accessed from the public domain. All searches were completed during the first week of September 2021. The distribution of the sample between various Australian universities is outlined in Table 2.

### Inclusion and exclusion criteria

All articles authored by Australian medical students in the AMSJ from 2010 to 2015 (Volumes 1–6) were retrospectively identified and analysed. Authors who graduated from medical school after 2016, or whose date of graduation was not identifiable were excluded. Only first authors were included. Authors whose university was not identifiable were excluded. The time frame was chosen to allow for students to graduate medical school and complete higher degrees or further research. All contributions to the AMSJ by Australian medical student were considered. Papers included in the analysis were classified as original research, literature reviews, and case reports. Other types of publications in the journal (editorials, letters to the editor, opinion pieces) were excluded from the analysis. For those universities without public online databases, the universities graduation office was emailed requesting access. If a database could not be accessed for data collection due to the university’s privacy policy, the student was excluded from the study.

### Outcome measures

For each AMSJ student author and control, the following data sets were gathered. The primary outcome measure was the number of PubMed® indexed citations which were searched using the PubMed® ‘author’ search tool and clarified using country affiliation identifiers. Secondary outcome measures included 1) the doctor’s current scope of clinical practice which was grouped into 3 categories: a) general practice or emergency medicine specialisation or training, b) surgeon or surgical training, c) physician or physician training. 2) The doctor’s attainment of higher education in the form of graduate certificate / diploma, Masters or PhD. 3) The doctor’s attainment of formal academic qualification in the form of conjoint / associate lecturer, researcher or professor.

### Data analysis

Data was organised into an Excel spreadsheet (Microsoft Corporation, Redmond Washington USA). The number of PubMed® indexed citations was recorded a both a whole number and a binary variable. All other outcome measures were recorded as binary variables (i.e. Masters Degree 1 or 0, PhD 1 or 0, Surgical Training 1 or 0 etc). All continuous variables were determined to have non-parametric distributions using the Shapiro-Wilk test. Data was loaded from the excel spreadsheet into the program ‘R®’ (version 4.1.1 The R Foundation for Statistical Computing) and logistic conditional regression was performed using the ‘clogit’ function. This was performed to express the independent association between variables as an odd ratio. A *p*-value < 0.05 was considered statistically significant.

## Results

### AMSJ authors

Eighty-six unique AMSJ authors were identified within the allocated time period. Thirty-one did not meet inclusion criteria, the reasons for which are summarised in Table 1. The remaining 55 student authors were included in the analysis. Articles included 14 case reports, 17 original research, 21 review articles. The majority of articles (98.1%) were published in the second half of the students’ degree or the year after graduation. Authors came from 14 different universities (see Table 2). Students’ genders were not public information and therefore unavailable.

**Table 1:** AMSJ authors that did not meet inclusion criteria

Reason Excluded	Number of Students
Graduated after 2016	6
Date of graduation unavailable	6
University unclear	6
University privacy policy preventing access to data	12 (from 5 universities)
Total	30

**Table 2:** Number of authors included in the sample from Australian universities after inclusion/exclusion criteria applied

University	Number of Students Included
Australian National University	1
Bond University	2
Deakin University	3
Flinders University	2
Griffith University	1
James Cook University	17
The University of Melbourne	3
Monash University	10
The University of Newcastle	1
The University of Notre Dame	2
The University of New South Wales	4
The University of Queensland	2
The University of Sydney	1
Wollongong University	6
**Total**	**55**

### PubMed® indexed citations

At the time of data collection, 37 of the 55 (67%) of student authors had achieved subsequent PubMed indexed publications. There was an average of 4.35 (1–19) PubMed indexed publications per author. This was compared to the control students of which 24 of the 110 (21%) achieved PubMed® indexed citations with an average of 3.9 (1–36) publications per author. Univariate analysis demonstrated that publication in the AMSJ was associated with future PubMed® indexed publications (OR 3.43, 95% CI 1.74–6.77, *P* < 0.001). These outcomes are summarised in Table 3.

**Table 3:** Univariate analysis for variables associated with AMSJ Publication

	Odds Ratio	95% CI	*P* value
Future PubMed® Indexed Publication	3.43	1.74–6.77	**< 0.001**
Any Higher Degree	4.05	1.99–8.22	**< 0.001**
Grad Cert / Diploma	3.54	0.82–15.31	0.091
Masters	3.31	1.50–7.30	**0.003**
PhD	2.11	0.65–6.86	0.213
Faculty Position	1.63	0.58–4.63	0.357
Specialty / Training Area			
Emergency / GP	1.11	0.51–2.38	0.792
Surgery	2.53	1.10–5.84	**0.029**
Physician	1.13	0.57–2.27	0.722

### Secondary outcome measures

Compared with controls, AMSJ authors were significantly more likely to obtain any higher degree than controls (OR 4.05, 95% CI 1.99–8.22, *P* = 0.0001). Subgroup analysis showed that this association was significant for Masters degrees (OR 3.31, 95% CI 1.50–7.30, *P* = 0.003) however was not significant for graduate certificates, diplomas or PhDs. AMSJ authors were also significantly more likely to enter into surgical training (OR 2.53, 95% CI 1.10–5.84, *P* = 0.029). There was no significant association with AMSJ publication and entry into general practice, emergency medicine or physician training. Additionally, there was no statistically significant association between AMSJ publication and future attainment of a faculty position (lecturer, researcher or professor). These outcomes are summarised in Table 2.

### Multivariate analysis

A multivariable conditional logistic regression model was created to account for potentially confounding variables. AMSJ authors were found to be more likely to achieve PubMed® indexed citations independently of whether they attained a higher degree or faculty position (OR 2.56, CI 1.22–5.39, *P* = 0.01). As expected, attainment of higher degree (OR 3.75, CI 1.71–8.22, *P* < 0.001) and faculty position (OR 9.77, CI 2.04–46.81, *P* = 0.004) were also independently predictive of PubMed® indexed citation. These results are summarised in Table 4.

**Table 4:** Multivariate conditional regression analysis model for variables associated with future PubMed® Indexed Publication

	Odds Ratio	95% CI	*P* value
AMSJ Publication	2.56	1.22–5.39	**0.013**
Higher Degree	3.75	1.71–8.22	**< 0.001**
Academic Position	9.77	2.04–46.81	**0.004**

## Discussion

Scientific publication of any kind during medical school has already been shown to be correlated with future publication and academic work [13–15]. Up until recently it has been unclear whether these findings extend to publication in medical student journals. Our study in many ways replicates the seminal work of Al-Busaidi et al. in New Zealand who used a similar conditional regression model to demonstrate that publication in a New Zealand MSJ (NZMSJ) is associated with higher rates of PubMed-indexed publications and higher degree attainment [5]. We have replicated this finding in an Australian cohort.

Al-Busaidi et al. additionally found a positive correlation between MSJ publication, PhD attainment and attainment of a faculty position. This was not the case in our cohort and may reflect differences between our nations’ medical education systems. New Zealand has only 2 medical schools each offering 6-year undergraduate MBBS courses. By contrast, Australia currently has 18 medical schools of which 14 are currently producing graduates. Medical degrees in Australia vary in length from 4 to 6 years with a mixture of post-graduate and under-graduate entry options [16]. At time of writing, 18 of these Australian medical schools offer their program as an MD. This presents a clear difference to the New Zealand cohort. Students within graduate entry programs tend to be older with a broader range of pre-medical experience. MD programs generally involve mandatory research components. These differences may have influenced our results, however studies have shown that course structure does not necessarily affect medical graduate performance or aptitude [17].

The retrospective design of this kind of study has certain limitations. The associations found may not indicate causation. Students who are interested in publishing research are more likely to continue publishing research after graduation [18]. The explicit goal of most student journals is to foster research enthusiasm and curiosity amongst medical students. While our study cannot explicitly prove that this goal is being met, we have strengthened the evidence that MSJs do actively engage with the very same individuals who become the academics of the future.

Despite a rigorous search strategy, there were limitations to our study’s data collection. A number of authors were excluded from the study due to lack of author affiliate information or universities without public databases. This may have influenced the results however the magnitude of this impact was not assessed. Five universities were unable to release data due to privacy policies, with student gender and age data largely unavailable. This was unfortunate given that a large body of literature reports a gender gap within academic research and it would have been useful to investigate whether this exists within the AMSJ [19–21]. Whilst the PubMed searches were rigorous, it was a challenge to attain accurate university data due to the large number of tertiary institutions and a lack of a centralised post-graduate database. As a result, Masters, PhD and faculty position data relied heavily on cross checking author-affiliate information and google search results. This may have introduced a source of information bias. Individuals who publish in student journals may possess a personality-type more likely to have a professional online presence which may have skewed the google search results. Certain specialties (i.e. surgeons) may also be more likely to advertise their postgraduate qualifications online than general practitioners or emergency specialists. These sorts of confounding variables may well have influenced our study’s findings. Finally, the sample size of our study is small, resulting in wide confidence intervals in some areas of the results. Whilst the size of our study is comparable to Al-Busaidi et al’s, future studies would benefit from a power calculation to compute a desired sample size in their methodology.

## Conclusion

We have shown that publication in a MSJ is associated with markers of academic success in an Australian cohort. PubMed®-indexed publications, attainment of a Masters degree, and entry in to surgical training were all significantly correlated to AMSJ publication. A conditional logistic regression model demonstrated that medical student publication in the AMSJ influences the number of future PubMed®-indexed publications, independent of major academic confounding variables.

## Supplementary Information

**Additional file 1.**

## Data Availability

Subsets of data are available from the corresponding author on reasonable request.

## Abbreviations

MBBS: Bachelor of Medicine, Bachelor of Surgery
MSJ: Medical student journal
MD: Doctor of Medicine
AMSJ: Australian Medical Student Journal
NZMSJ: New Zealand Medical Student Journal
GP: General practitioner
PhD: Doctor of Philosophy

## References

[1] Stringer MD, Ahmadi O (2009). Famous discoveries by medical students. ANZ J Surg.

[2] Al-Busaidi IS (2016). Medical student journals: critical to the development of physician-scientists. Educ Health (Abingdon).

[3] Alamri Y (2016). How do medical student journals fare? A global survey of journals run by medical students. Educ Health (Abingdon).

[4] Al-Busaidi IS, Alamri Y (2018). Peer review policies in medical student journals. Postgrad Med J.

[5] Al-Busaidi IS, Wells CI, Wilkinson TJ (2019). Publication in a medical student journal predicts short- and long-term academic success: a matched-cohort study. BMC Med Educ.

[6] Traill CL (2016). Time to research Australian physician-researchers. Intern Med J.

[7] Windsor J (2017). The clinical academic workforce in Australia and New Zealand: report on the second binational summit to implement a sustainable training pathway. Intern Med J.

[8] Windsor J (2015). Building a sustainable clinical academic workforce to meet the future healthcare needs of a ustralia and N ew Z ealand: report from the first summit meeting. Intern Med J.

[9] Eley DS (2018). The clinician-scientist track: an approach addressing Australia’s need for a pathway to train its future clinical academic workforce. BMC Med Educ.

[10] Amgad M (2015). Medical student research: an integrated mixed-methods systematic review and Meta-analysis. PLoS One.

[11] Wells C (2016). Rate and predictors of publication by medical and health science summer research students: a 14-year analysis from Auckland, New Zealand. MedEdPublish.

[12] Australian Medical Student Journal. 2021 10/10/2021]; Available from: http://www.amsj.org/about/aims.

[13] Fang D, Meyer RE (2003). Effect of two Howard Hughes Medical Institute research training programs for medical students on the likelihood of pursuing research careers. Acad Med.

[14] Evered DC (1987). The correlates of research success. Br Med J (Clin Res Ed).

[15] Brancati FL (1992). Early predictors of career achievement in academic medicine. Jama.

[16] Australian Government, D.o.H (2019). Medical Education and Training Data Set.

[17] Garrud P, McManus IC (2018). Impact of accelerated, graduate-entry medicine courses: a comparison of profile, success, and specialty destination between graduate entrants to accelerated or standard medicine courses in UK. BMC Med Educ.

[18] Alamri Y, Monasterio E, Wilkinson TJ (2021). Factors Predictive of Medical Student Involvement in Research: Results from a New Zealand Institution. Adv Med Educ Pract.

[19] Mueller C, Wright R, Girod S (2017). The publication gender gap in US academic surgery. BMC Surg.

[20] Okike K (2012). The orthopedic gender gap: trends in authorship and editorial board representation over the past 4 decades. Am J Orthop (Belle Mead NJ).

[21] Wells C, Al-Busaidi I (2017). Student publications in the New Zealand medical student journal: the first fourteen years. New Zealand Med Stud J.

